# Spinal bone metastases in colorectal cancer: a retrospective analysis of stability, prognostic factors and survival after palliative radiotherapy

**DOI:** 10.1186/s13014-017-0852-6

**Published:** 2017-07-11

**Authors:** Tilman Bostel, Robert Förster, Ingmar Schlampp, Tania Sprave, Thomas Bruckner, Nils Henrik Nicolay, Stefan Ezechiel Welte, Jürgen Debus, Harald Rief

**Affiliations:** 1 Department of Radiation Oncology, University Hospital Heidelberg, Im Neuenheimer Feld 400, 69120 Heidelberg, Germany; 2 Department of Medical Biometry, University Hospital Heidelberg, Heidelberg, Germany; 3 National Center for Radiation Oncology (NCRO), Heidelberg Institute for Radiation Oncology (HIRO), Im Neuenheimer Feld 400, 69120 Heidelberg, Germany

## Abstract

**Background:**

This retrospective analysis aimed to analyse the stability of spinal bone metastases in colorectal cancer (CRC) patients following radiotherapy (RT) by use of a validated score and to assess prognostic factors for stability and survival.

**Methods:**

Ninety-four patients with osteolytic spinal bone metastases from CRC were treated at the Department of Radiation Oncology at the University Hospital Heidelberg between 2000 and 2014. The stability of each affected vertebral body was assessed according to the validated Taneichi bone stability score on the basis of the treatment planning CT scan prior to RT and also based on the follow-up CT examinations at 3 and 6 months after RT. Additionally, bone survival rates (time between first day of RT and death from any cause) as well as prognostic factors for bone survival were evaluated for all study patients.

**Results:**

Before RT, 59 patients (63%) were rated unstable according to the Taneichi score. Pathological fractures within the irradiated region were diagnosed in 43 patients (46%) prior to RT. New fractures or progression of previously collapsed vertebrae were diagnosed in 4 patients (4%) after irradiation. Significant re-calcification and stabilization of former unstable bone metastases was only observed in 3/59 patients (3%) and 5/59 patients (9%). The median bone survival was 4.2 months (range 0.5–67.3 months) and 6 months after RT 61% of the patients were dead. Karnofsky performance score (KPS) (< 70% vs. ≥ 70%), chemotherapy and bisphosphonate therapy were predictive prognostic factors for bone survival.

**Conclusions:**

Our study population is characterized by poor bone survival and low re-calcification rates of unstable spinal bone lesions 3 and 6 months after RT. To avoid unnecessary hospitalisation and improve remaining QoL, short fractionated treatment schedules of RT may be prefered in this highly palliative situation, particularly for patients with a KPS < 70%.

## Background

Colorectal cancer (CRC) is one of the most common cancers worldwide, with more than 1 million new cases per year [1]. While the prognosis of CRC has improved over the last decade with the advent of neoadjuvant treatment regimes and the introduction of targeted agents, the metastatic form of CRC remains to have a poor prognosis with a 5-year survival rate of only 10% [1]. It is well known that the most common target sites for metastatic spread are the liver and the lungs [2–4]. Skeletal involvement is also a relative frequent finding in metastatic CRC, and is mostly associated with distant metastases in other organs such as liver or lung [5–7]. Up to 5, 5% of all CRC patients have bone metastasis at the time of initial diagnosis and up to 27% will develop bone metastasis during the course of their disease [2, 5, 6]; the most common localization of bone metastases is the vertebral column [8]. Due to novel treatment approaches for patients with metastatic CRC, the median survival of affected patients has increased significantly, and as a consequence, patients have a higher risk not only to develop bone metastases but also to experience complications arising from metastatic bone destruction, such as pain, pathological fractures, spinal cord compression or hypercalcemia [9–16]. These skeletal-related events have the potential to severely affect patients’ quality of life (QoL) [17].

The treatment of bone metastases requires a multidisciplinary approach employing surgery, systemic treatment or radiotherapy [18]. Pain, existing or impending instability, neurological symptoms due to compression of the spinal cord, or previous surgical intervention form the main indications for radiotherapy (RT). RT offers pain relief in 50–80% of patients [19].

Besides pain, impaired stability of metastatic vertebral bodies can result in severe complications and therefore strongly affect patients’ QoL. Insufficient stabilization of the metastatic vertebral column may lead to severe disability from pathologic fractures; however, commonly prescribed surgical corsets add a significant immobilization to already existing pain symptoms. Recently, we reported on patients with lung cancer, breast cancer and pelvic gynecologic malignancies in which a significant response towards RT in terms of stability could be demonstrated [20, 21, 22]. The benefit of RT concerning pain and recalcification of osteolytic metastases may be increased when the therapy is combined with concomitant administration of bisphosphonates [23, 24].

The aim of this retrospective analysis was to systematically assess the bone lesions resulting from CRC in terms of stability, fractures before and after RT, survival, and predictive factors for stability and survival.

## Methods

Ninety-four patients with bone metastases of the thoracic and lumbar spine resulting from CRC were treated at the Department of Radiation Oncology at Heidelberg University Hospital between February 2000 and July 2014. Patients’ data were taken from the cancer registry of the National Center for Tumour Diseases (Heidelberg, Germany). Patients underwent regular follow-up examinations including computed tomography (CT) imaging. Diagnosis was based on CT, magnetic resonance imaging (MRI) or bone scintigraphy findings. The evaluated osteolytic bone metastases had to be located in the thoracic or lumbar spine.

The stability of each affected vertebral body was assessed according to the validated Taneichi bone stability score on the basis of the treatment planning CT scan prior to RT and also based on the follow-up CT examinations at 3 and 6 months after RT [25]. This scoring system constitutes a simple method for classifying osteolytic metastases in vertebral bodies as “stable” or “unstable” by definition of risk factors such as tumor size and the degree of costovertebral joint destruction for the thoracic region (Th 1 to 10) and tumor size and the degree of pedicle destruction for the lumbar region (Th 11 to L5).

**Fig. 1:**
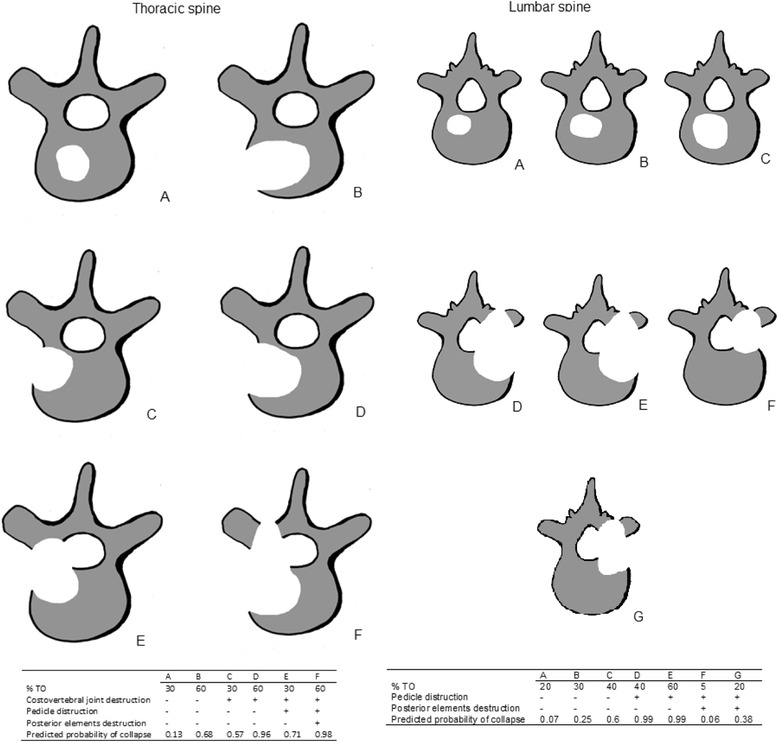
Taneichi score

Osteolytic metastases were rated on a scale from A to G, whereby subtypes A to C were defined as stable, and subtypes D to G as unstable (Fig. 1). In patients with more than one metastasis per vertebral body, the localization with the highest Taneichi score was assessed.

Many patients (43%) exhibited more than one affected vertebral body within the planning target volume (PTV). Accordingly, 94 patients presenting with a total of 162 bone lesions in the thoracic and lumbar spine were evaluated. The Karnofsky performance score (KPS) was used to assess performance status [26]. Many patients received further treatments such as chemotherapy or bisphosphonates before, during and after radiotherapy. The characteristics of all patients included in this study are summarized in Tables 1 and 2.

**Table 1:** Patients’ characteristics

		*n*	%
Age (years)			
Median (range)	66 (43–88)		
Gender			
Female		35	37.2
Male		59	62.8
Karnofsky PS			
< 70%		54	57.4
≥ 70%		40	42.6
Number of bone metastases			
Mean (range)	1.7 (1–5)		
Solitary		54	57.4
Multiple		40	42.6
Spine involvement			
Thoracic		56	60
Lumbar		38	40
Primary site			
Cecum		4	4.3
Ascending, transverse and descending colon		27	28.7
Sigma		18	19.1
Rectum		45	47.9
Distant extraskeletal metastases			
Brain		13	13.8
Lung		61	64.9
Liver		72	76.6
Lymphatic		35	37.2
Others		22	23.4

*Abbreviation: Karnofsky PS* Karnofsky performance score

**Table 2:** Treatment

Characteristics	*n*	%
RT dose completed (Gy)		
Single dose (median, range)	3	(2–4)
Cumulative dose (median, range)	30	(20–40)
Treatment for primary site		
Chemotherapy	80	85.1
Targeted therapy	26	27.7
Other treatment for bone metastases		
Orthopedic corset	27	28.7
Bisphosphonates	21	22.3

*Abbreviations: RT* radiotherapy*, Gy* Gray

RT was planned on the basis of a planning CT examination and performed over a dorsal photon field with the energy 6 MV. PTV covered the affected vertebral body/bodies as well as the vertebral body immediately above and below. Median delivered dose was 30 Gy (range 20–40 Gy) in individual treatment fractions of 3 Gy (2–4 Gy) (Table 2).

Statistical analysis was done using the SAS software version 9.3 (SAS Institute, Cary, NC, USA). A *p*-value of *p* < .05 was considered statistically significant (Chi square and Log-rank test). Bone survival was defined as the time between first day of RT for bone metastases until death from any cause. Survival was plotted according to the Kaplan-Meier method. Bowker’s test and kappa statistics were calculated to evaluate distribution of the Taneichi score over time. Univariate logistic regression analysis was performed to evaluate possible predictors for bone survival.

This analysis was approved by the independent ethics committee of the Heidelberg University Medical Faculty (# S-513/2012).

## Results

The mean follow-up time was 7.5 months (range 0.5–67.3 months). Ninety-four patients with a total of 162 bone metastases (range 1–5 metastases/ patient) were assessed according to the validated Taneichi scoring system prior to RT and at 3 and 6 months after RT based on CT imaging. Osseous metastases were located in the thoracic spine in 60% (*n* = 56) and in the lumbar spine in 40% (*n* = 38) of patients. Pathological fractures within the irradiated region were diagnosed in 43 patients (46%) prior to RT. New fractures or progression of previously collapsed vertebrae were diagnosed in 4 patients (4%) after irradiation.

The most frequently observed Taneichi subtype was D (31%; *n* = 29) (Fig. 1). None of the initially stable bone lesions were rated unstable during the course of follow-up (Table 3).

**Table 3:** Results of Taneichi score evaluation

	*n*	%
Stability before RT		
Unstable	59	63
Stable	35	37
Stability after 3 months		
Unstable	27	59
Stable	19	41
Stability after 6 months		
Unstable	17	46
Stable	20	54

*Abbreviation: RT* radiotherapy

In contrast, RT could stabilize primary unstable bone metastases in 5 and 9% of patients after 3 and 6 months (*p* = 0.08 and 0.03, McNemar test) (Table 3). In patients with KPS ≥ 70%, the stabilization rate was higher than in patients with KPS < 70% after 6 months (16% vs. 0%). Due to relatively low stabilization rates, the planned analysis of predictive factors for stabilization was not possible. Taneichi subtypes improved in 49% (*n* = 18) and showed no change in 51% (*n* = 19) of the patients who were still alive 6 months after RT. No deterioration in the scored Taneichi value was observed at the follow-up examinations compared to the baseline CT scan. The Bowker test shows the distribution pattern of the subtypes according to the Taneichi score prior to and 3 and 6 months after RT (Tables 4 and 5). Asymmetry was apparent and correlation was good (weighted kappa = 0.85 and 0.63, Tables 4 and 5).

**Table 4:** Test of symmetry for Taneichi score (3 months)

Subtypes 3 months after RT									
		A	B	C	D	E	F	G	Total
Subtypes before RT	A	2	0	0	0	0	0	0	2
	B	1	6	0	0	0	0	0	7
	C	0	3	4	0	0	0	0	7
	D	1	0	1	13	0	0	0	15
	E	0	0	1	1	7	0	0	9
	F	0	0	0	0	0	4	0	4
	G	0	0	0	0	0	0	1	1
	Total	4	9	6	14	7	4	1	45

This Bowker Test shows the distribution of subtypes of Taneichi-Score before and 3 months after RT. The evaluation of the distribution of subtypes A to G shows in some patients minor changes in the direction of improvement over the course of time. Deterioration occurs in no cases, improvement in 18% (*n* = 8). No change is seen in 82% (*n* = 37) of the patients who were still alive more than 3 months after RT

*Abbreviation: RT* radiotherapy

**Table 5:** Test of symmetry for Taneichi score (6 months)

Subtypes 6 months after RT									
		A	B	C	D	E	F	G	Total
Subtypes before RT	A	2	0	0	0	0	0	0	2
	B	5	1	0	0	0	0	0	6
	C	3	3	1	0	0	0	0	7
	D	1	0	3	9	0	0	0	13
	E	1	0	0	1	3	0	0	5
	F	0	0	0	0	1	2	0	3
	G	0	0	0	0	0	0	1	1
	Total	12	4	4	10	4	2	1	37

This Bowker Test shows the distribution of subtypes of Taneichi-Score before and 6 months after RT. Deterioration of Taneichi-subtypes occurs in no cases, improvement in 49% (*n* = 18). No change is seen in 51% (*n* = 19) of the patients who were still alive more than 6 months after RT

*Abbreviation: RT* radiotherapy

**Fig. 2:**
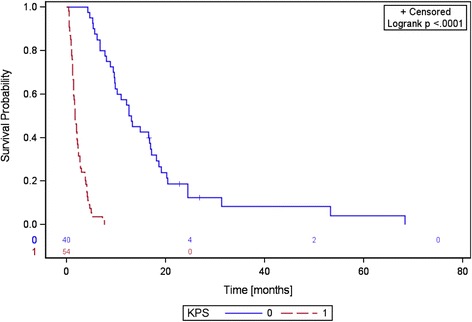
Bone survival depending on the Karnofsky performance score (KPS)

Thirty-seven patients (39%) were still alive at 6 months after RT. The median bone survival for the entire patient cohort was 4.2 months (range 0.5–67.3 months). KPS was a strong predictive factor for bone survival (*p* < 0.0001) (Fig. 2). Median bone survival was 1.7 months (range 0.5–7.5 months) for patients with a KPS < 70% in contrast to 12.4 months (range 4.3–67.3 months) for patients with a KPS of ≥70%. The use of bisphosphonates and chemotherapy were further predictive factors significantly correlated with bone survival in the univariate analysis (Table 6). The prevalence of additional visceral metastases, the use of targeted agents and the number of bone metastases were not predictive for bone survival (Table 6).

**Table 6:** Results of prognostic factors related to bone survival

Parameter	*p*-value	HR	CL
Karnofsky PS (≥ 70% vs. < 70%)	< 0.0001	26.85	10.95–65.87
Chemotherapy (yes vs. no)	< 0.01	0.49	0.29–0.84
Targeted agents (yes vs. no)	n.s.	0.83	0.43–1.6
Bisphosphonate therapy (yes vs. no)	< 0.05	0.5	0.28–0.89
Visceral metastases (yes vs. no)	n.s.	2.07	0.88–4.9
Number of bone metastases (1 vs. > 1)	n.s.	1.32	0.83–2.1

*Abbreviations: Karnofsky PS* Karnofsky performance score*, HR* Hazard Ratio*, CI* Confidence Limits of the results for a confidence level of 95%*, n.s.* not significant

## Discussion

The major problems of patients with bone metastasis that commonly reduce quality of life comprise severe, drug-resistant pain symptoms, manifest or impending fractures, tumor-induced hypercalcemia and neurological complications such as paraplegia.

Therefore, classification of stability of spinal metastases is a frequent clinical concern. The Taneichi scoring system is an established tool for the classification of spinal metastases regarding risk of pathological fracture or bone instability.

In previous studies, therapeutic effects of palliative RT on colorectal spinal metastases were only measured in terms of pain control and improvement of neurological deficits due to metastatic spinal cord compression [27]. In contrast, there are no existing data concerning the impact of RT on the stability of spinal metastases due to colorectal cancer.

Prior to RT, 63% of the patients in this dataset had unstable bone metastases of the thoracic or lumbar spine. After 6 months, palliative RT reached significant re-ossification and stabilization in only 9% of these bone lesions.

The reported stabilization rate is relatively poor compared to the stabilization rate of other tumor entities after palliative RT such as lung or breast cancer and pelvic gynecological malignancies [19, 22, 23]. In contrast, spinal metastases from malignant melanoma and renal cell carcinoma do not benefit at all from palliative RT with regard to re-calcification and stabilization of unstable spinal bone lesions [28, 29].

In our study, the poor stabilization effect of palliative RT can be explained to a large extent by the limited life expectancy of the patients. At 6 months after RT, only 39% of patients were still alive. Median survival was 4.2 months after diagnosis of bone metastasis and corresponds well with the results of previously reported patient cohorts [6, 30, 31]. The very poor bone survival of CRC patients is explained by the fact that bone metastasis from CRC represents a late event in the evolution of the disease, with the majority of the patients exhibiting widely disseminated disease at the time of first diagnosis of skeletal manifestation [6, 7].

In line with the results of another study [27], we identified the patients’ performance status as a prognostic factor for predicting bone survival in CRC patients. A Karnofsky performance status (KPS) < 70% was significantly associated with an extremely poor bone survival of less than 2 months. In contrast, patients with a KPS ≥ 70% had a bone survival of about 12 months. As recalcification of irradiated osteolytic bone lesions usually takes up to several months, it is unlikely that patients with a KPS < 70% will have a benefit in terms of stabilization. In contrast, patients with a KPS ≥ 70% have a higher chance for stabilization due to longer life expectancy and continued mobility-related physical strain to the bones. In our cohort, 4 out of 25 patients (16%) with initially unstable bone lesions and a KPS ≥ 70% were found stabilized by RT within 6 months.

Additionally, our results showed that patients with chemotherapy and/or bisphosphonate therapy had an improved bone survival compared to patients without these therapies. In 81% of patients, bisphosphonates were initiated after the end of radiotherapy. In contrast, chemotherapy was started prior to RT in 93% of patients.

In contrast, the absence or presence of visceral metastases, the number of bone metastases and the use of targeted agents were not statistically significant in the univariate analysis.

Our study has several limitations, among them the retrospective character of the dataset. Secondly, due to the limited number of patients, a statistically sound multivariate analysis of prognostic factors for bone survival was not possible. Additionally, the selection criteria for the application of chemotherapy and bisphosphonates were retrospectively not available; therefore, we cannot rule out that the use of both treatment modalities may be related to a better performance status rather than being an independent predictive factor for bone survival. A planned analysis of prognostic factors for stabilization of initially unstable bone metastases was not possible due to the poor bone survival and low bone recalcification rates at 3 and 6 months after RT. Due to the limitations of the Taneichi score, patients with bone metastases from CRC outside the thoracic and lumbar spine were not considered.

Summarized, due to the low re-calcification rates observed in our patient cohort, short course RT appears preferable for patients with a KPS < 70%, since it provides similar pain control as protracted schedules. In contrast, patients with a KPS ≥ 70% may be treated with more protracted radiation schedules, if the treatment goal is stabilization, even though the chance for attaining this objective is relative small compared to other tumor entities [22, 23].

## Conclusion

Patients with bone metastases due to CRC exhibit poor bone survival and low re-calcification rates of unstable spinal bone lesions at 3 and 6 months after RT. To avoid unnecessary hospitalisation and improve QoL, short fractionated treatment schedules of RT may be preferable in this highly palliative situation, particularly for patients with a KPS < 70%.
